# Short-time anesthesia of a child with phenylketonuria: a case report

**DOI:** 10.1016/j.bjane.2021.04.013

**Published:** 2021-04-28

**Authors:** Masoud Tarbiat, Sayed Ahmad Reza Salimbahrami, Ali Goudarzi, Mahmoud Rezaei

**Affiliations:** a Hamadan University of Medical Sciences, School of Medicine, Department of Anesthesiology, Fellowship of Cardiac Anesthesia, Hamadan, Iran; c Hamadan University of Medical Sciences, School of Medicine, Department of Orthopedic Surgery, Hamadan, Iran; d Hamadan University of Medical Sciences, School of Medicine, Department of Anesthesiology, Hamadan, Iran

**Keywords:** Anesthesia, Phenylketonuria, Ketamine, Midazolam

## Abstract

Phenylketonuria is a rare disorder that increases the levels of phenylalanine in the blood. As there are scant articles about anesthesia management in phenylketonuria patients, this encouraged us to report a short-time anesthesia management of a child with phenylketonuria for bone fracture. The anesthesia was induced with intravenous ketamine and midazolam. During procedure, he received 100% oxygen via a face mask throughout spontaneous breathing. The operation was uneventful, and he was completely awakened in the recovery room. This report emphasizes that in some situations, the combination of midazolam with ketamine could be used safely for short time anesthesia in phenylketonuria patients.

## Introduction

Phenylketonuria (commonly known as PKU) is an autosomal recessive disorder due to the deficient or defective of phenylalanine hydroxylase enzyme. This disorder is associated with abnormal accumulation of the levels of phenylalanine amino acid and its metabolites in the body. The occurrence of PKU is 1:10,000–30,000 newborns worldwide.^1^, ^2^, ^3^ These patients are a challenge to anesthesiologists for abnormal metabolic pathways and their consequences.

In the review, scant articles have been found about anesthesia management in these patients (specially affected children). Therefore, this encouraged us to share our experience about a child with PKU disorder who had anesthesia for a bone fracture.

## Case report

A 4-year-old child diagnosed with PKU in the neonatal period was admitted to our hospital for treatment of a first metatarsal bone fracture in his left foot. Apart from phenylalanine-restricted diet for PKU, he took no medication. He had no history of seizure, mental deficiency, microcephaly, and cardiac defects. He also had no symptom, and the physical examination was normal. All routine laboratory investigations were normal and the arterial blood sample revealed a pH of 7.49, PaCO_2_ of 20.1 mmHg, NaHCO_3_ of 15.4 mEq/liter and PaO_2_ of 87.4 mmHg on room air. After consultation with a pediatrician, he was transported to operation theatre for bone fracture repair. In the operating room, he was monitored via standard monitoring and his vital signs were as follows: blood pressure of 105/61 mmHg, heart rate of 101 beats/min with sinus regular rhythm and respiratory rate of 18 breaths/min. His oxygen saturation (SPO_2_) was 97 on room air. After preoxygenation with 100% oxygen, anesthesia was induced with intravenous midazolam 1.5 mg, ketamine 15 mg and atropine 0.25 mg. During closed reduction and splinting procedures, he received 100% oxygen via an oxygen face mask throughout spontaneous breathing. The operative course (10 minutes) was uneventful, and he was completely awakened 20 minutes later in the recovery room.

## Discussion

Phenylalanine is an essential amino acid. It exists in all proteins and in some artificial sweeteners. Phenylalanine hydroxylase enzyme converts phenylalanine amino acid to tyrosine. The deficiency of phenylalanine hydroxylase results in accumulation of phenylalanine and its metabolites in the body. The Phenylalanine Hydroxylase (*PAH*) gene provides instructions for making an enzyme called phenylalanine hydroxylase. The *PAH*gene is located on chromosome 12q23.2. It is responsible for the first step in processing phenylalanine. Phenylketonuria is an autosomal recessive inherited disorder that due to the deficiency of *PAH* enzyme. It is usually characterized by elevations in plasma phenylalanine level more than 1200 μmoL.L^-1^ (> 20 mg.dL^-1^). Therefore, the clinical basis of the disease is accumulation of phenylalanine and lack of tyrosine in the body. The brain is the main organ damaged by PKU. The symptoms and signs of PKU are seizures, psychiatric disorders, skin disorders (eczema, seborrheic rash), musty odor, microcephaly, hyperreflexia, hypertonia, autism, and mental retardation. The mainstay of treatment of phenylketonuria is a low-phenylalanine diet.^1^, ^3^ At present, there is no evidence to recommend specific anesthesia management for PKA patients. However, some physicians reported their experience in anesthesia management of them.

Matsushita and colleagues described general anesthesia for dental caries treatment for an intellectually disabled adult woman with PKU who also had mental retardation and epilepsy. They induced general anesthesia with propofol, fentanyl, and rocuronium. For maintenance of anesthesia, propofol-remifentanil infusion and nitrous oxide were used. In perioperative care, the patient had no serious neurological symptoms.^4^ Torun and his associate reported a severe bronchospasm in a child with phenylketonuria during general anesthesia. They induced general anesthesia with intravenous propofol, fentanyl, and vecuronium. The sevoflurane and a mixture of 50% oxygen and 50% air were used for maintenance of anesthesia. The bronchospasm occurred immediately after intubation. The bronchospasm was treated successfully with inhaled salbutamol and intravenous methylprednisolone. Since catecholamines are synthesized through a series of reactions commenced by tyrosine, and it is required for synthesis of catecholamines. Therefore, synthesis of catecholamines may be diminished in PKU patients for the absence of tyrosine. Eventually, they concluded that the bronchospasm was related to lack of catecholamines induced by phenylketonuria.^3^ In another case report, Lee et al. noted a spastic paraparesis in a 14-year-old male with PKU who had general anesthesia with nitrous oxide for myringoplasty. They opined that the spastic paraparesis was due to vitamin B12 deficiency and restricted consumption of vitamin B12-containing foods in phenylketonuria. Therefore, they suggested that nitrous oxide anesthesia has potential risk in PKU patients.^5^ Rayadurg and colleagues also described tachycardia, fever, and severe tachypnea (signs of propofol infusion syndrome) during sedation with intravenous bolus (10 mg) and infusion (12 mg.h^-1^) of propofol in a 1-year-old child with PKU for Magnetic Resonance Imaging (MRI). Because phenylketonuria and propofol inhibits the activity of mitochondrial complex I by competing with nicotinamide adenine dinucleotide phosphate, they recommend avoiding the administration of propofol for sedation or general anesthesia in these patients.^2^ In our case, since the procedure was supposed to take less than a few minutes (< 15 min), it was decided to use midazolam and ketamine. Midazolam is a short-acting benzodiazepine (act selectively at the GABA A receptor) used for premedication, sedation, and anesthesia. The onset of action of midazolam is usually rapid (usually peak effect reached within 2 to 3 minutes). It increases the seizure initiation threshold and has neuroprotective effects by preventing lipid peroxidation and mitochondrial damage. Midazolam attenuates the hemodynamic effects (like tachycardia and hypertension) of ketamine. Ketamine (Ketalar) was synthesized in 1962 by Stevens. It produces dissociative anesthesia (dose-related unconsciousness and analgesia). Ketamine acts at multiple receptors including the NMDAR (N-methyl-D-aspartate receptor), opioid receptors, and monoaminergic receptors. Ketamine also produces undesirable psychological reactions (vivid dreaming, sense of floating out of body, and illusions). Ketamine has an onset of action within 30 to 60 seconds of intravenous administration.

The combination of ketamine with midazolam eliminates or attenuates the undesirable hypertension and tachycardia, and postoperative psychological disturbances.

## Conclusion

This case demonstrates that the use of a combination of ketamine and midazolam for the anesthesia management of children with phenylketonuria could be feasible and safe. However, further studies could help establish optimal anesthesia management for these patients.

## Informed consent

The patient’s parents signed informed consent for the publication of this article.

## Conflicts of interest

The authors declare no conflicts of interest.
